# NETWORK ANALYSIS OF ANXIETY AND DEPRESSIVE SYMPTOMS AMONG OLDER ADULTS WITH DIFFERENTIAL MENTAL HEALTH RISKS

**DOI:** 10.1093/geroni/igad104.3036

**Published:** 2023-12-21

**Authors:** Tianyin Liu, Wen Zhang, Gloria Hoi-Yan Wong, Terry Lum

**Affiliations:** Tthe University of Hong Kong, Hong Kong, Hong Kong; Hong Kong Metropolitan University, Hong Kong, Hong Kong; Hong Kong Metropolitan University, Hong Kong, Hong Kong; Tthe University of Hong Kong, Hong Kong, Hong Kong

## Abstract

Anxiety and depressive symptoms are common in older people, and network analysis may provide deeper characterization of symptom-symptom interactions to inform personalized care. The aim of this study was to elucidate characteristics of anxiety and depressive symptom networks of older adults with differential risks for depression and anxiety. A total of 4184 older adults (mean age = 77.3±8.8 years, 3245 women) were recruited from the community. Their anxiety and depressive symptom were measured using the Generalized Anxiety Disorder Scale 7-item (GAD-7) and Patient Health Questionnaire 9-item (PHQ-9), respectively. A cut-off score of 5 was used for both scales to differentiate people with mild or above anxiety/depressive symptoms; by this criterion, 1067 (25.5%) of the sample had no anxiety/depressive symptoms, 1690 (40.4%) had symptoms in one domain, and 1427 (34.1%) had symptoms in both domains. Central symptoms and bridge symptoms were identified via centrality indices and bridge centrality indices, respectively. Network stability was examined using the case-dropping procedure. Overall, Restlessness (GAD-7 item 5), Depressed mood (PHQ-9 item 2), and Fear (GAD-7 item 7) had the highest centrality values; two bridge symptoms, Restlessness and Psychomotor disturbances (PHQ-9 item 8), were also identified. Higher risks for depression and/or anxiety were positively associated with global strength; Restlessness and Depressed mood remained to be central symptoms, but in those with both depression and anxiety risks, Restlessness and Guilt/Self-blame (PHQ-9 item 6) bridged the two domains. Interventions designed to target central symptoms and bridge symptoms may be effective in alleviating co-occurring experiences of anxiety and depression.

